# Problematic trading: a Systematic Review of theoretical considerations

**DOI:** 10.3389/fpsyt.2025.1505012

**Published:** 2025-04-29

**Authors:** Yura Loscalzo, Guyonne Rogier, Patrizia Velotti

**Affiliations:** ^1^ Department of Health Sciences, University of Florence, School of Psychology, Florence, Italy; ^2^ Department of Dynamic and Clinical Psychology, and Health Studies, Sapienza University of Rome, Rome, Italy; ^3^ Saint Camillus International University of Health and Medical Sciences, UniCamillus University, Rome, Italy

**Keywords:** addiction, crypto-currency, pathological trading, problematic trading, stock market, theory

## Abstract

**Background and aims:**

Recently, the psychological literature increased attention to problematic financial trading, usually adopting the behavioral addiction framework aprioristically. Therefore, we aim to systematize the theoretical literature across different scholarly areas to detect if there is an accurate theorization and operationalization of the construct that considers the features of problematic trading.

**Methods:**

We used six scientific databases (MEDLINE, PsycARTICLES, PsycINFO, PubMed, Scopus, and Web of Science). We found that 23 papers met our eligibility criteria.

**Results:**

Our systematic review showed that problematic trading received attention since the ‘90s in the economy/law area. However, none of the 11 non-psychological papers conceptualized problematic trading as a phenomenon on its own. Regarding instead the psychological literature, ten up to 12 papers used the behavioral addiction framework, usually assimilating problematic trading to gambling. Moreover, only four papers conceptualized it as a distinct diagnosis from gambling.

**Discussion and conclusions:**

Even if the psychological literature seems to begin supporting the conceptualization of problematic trading as a disorder on its own, there is a tendency to assimilate it into gambling. We recommend that future studies analyze problematic trading as a distinct phenomenon and avoid the aprioristic use of the gambling framework to unveil the features of this new potential clinical disorder. Thus, qualitative studies for in-depth knowledge of problematic trading would be critical before suggesting a specific operationalization of the construct and a scale of measure.

**Systematic review registration:**

https://www.crd.york.ac.uk/PROSPERO/view/CRD4202455828, identifier CRD42024558280

## Introduction

In the last decade, the psychological literature devoted significant attention to problematic/excessive trading, usually adopting the behavioral addiction framework, with specific reference to Gambling Disorder (e.g., 1–7). Considering that the literature highlighted that gambling and financial markets share common features (8, 9), as well as taking into account the recent tendency for the “gamblification” of financial products (9–11), scholars showed a propensity to adopt the gambling/behavioral addiction framework for describing excessive and impairing trading. In line with this, Lee et al. (12) recently conducted a systematic review of the association between gambling and financial trading, starting from the consideration that gambling literature generally addressed traditional gambling activities, such as poker, lotteries, and sports betting, but that gambling has always existed also in financial markets (13, 14). They showed, through the analysis of 12 studies, that financial trading (in the form of stock trading, day trading, and cryptocurrency trading) is associated with a higher risk for problem gambling, especially for those engaged in speculative trading behaviors like day trading and cryptocurrency trading (12). Also, Lee et al. (12) showed that the prevalence of problem gambling in people involved in financial trading is higher than the prevalence generally found for problem gambling. Therefore, it is not surprising that – well before the widespread interest in problematic trading – Shaffer and Freed (15) included financial markets among the list of gambling activities without specifying which type of specific trading behaviors this label included and the scientific evidence supporting the inclusion of financial activities among gambling-related disorders.

However, the psychological literature warns against using the addiction framework aprioristically. Billieux et al. (16) underscored that 2013 – the year of publication of the DSM-5 (17) – testified a peak of 2563 papers concerning various behavioral addictions, also about common behaviors such as tanning and dancing (18, 19). The inclusion of Gambling Disorder in the addictive behaviors section of DSM-5 (17) – and, therefore, its official recognition as a behavioral addiction – might have incentivized scholars to analyze many excessive behaviors through the lens of the addiction perspective. Though, as cautioned by Billieux et al. (16), scholars often adopted an atheoretical and confirmatory approach, where they used aprioristically the addiction framework with the related risk of over-pathologizing common behaviors. In the same line, Kardefelt-Winther (20) stressed that scholars, when proposing new potential behavioral addictions, should go beyond the addiction model to discover the actual manifestation of the problematic behavior. Therefore, even if there seems to be consensus in the psychological literature about the use of the gambling framework in the analysis of excessive trading, it is of critical value systematizing the theoretical literature concerning the topic (including contributions from areas other than psychology) to unveil if there is an accurate operationalization of the construct – which might then be used by different scholars to promote a cumulative knowledge of the phenomenon. In line with this, even if referring to the workaholism field, Quinones and Griffiths (21) pinpointed the importance of developing an accurate theorization before creating a new construct and related instruments (instead of merely applying, in the case of problematic trading, gambling criteria).

In conclusion, having a proper definition of problematic trading is of vital importance, given that trading is a risky practice involving financial losses, which is widespread also thanks to the fact that, nowadays, anyone can easily open an online account and invest in international marketplaces (2) as a consequence of the changes in investment methods that arose with Internet development (22). Thus, our study aims to systematize the theoretical literature about problematic trading across different scholarly areas, to detect if there is an accurate theorization and operationalization of the construct of problematic trading which did not apply aprioristically the behavioral/gambling framework and take instead into account the features of problematic trading.

## Method

**Figure 1:**
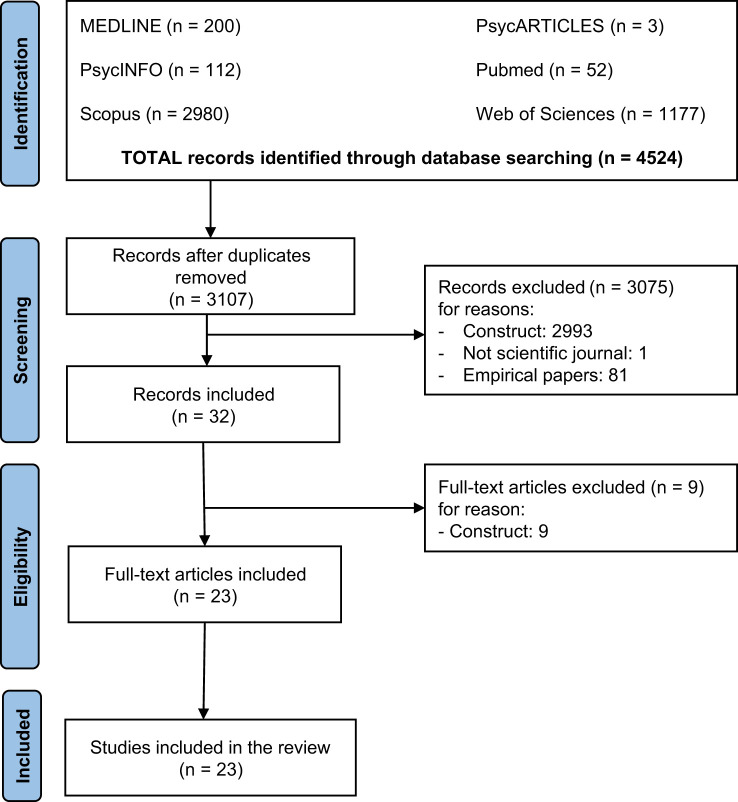
Flow diagram.

We conducted a systematic search in line with PRISMA guidelines (23) and the Population, Intervention, Comparison, Outcomes, and Study framework (24). **Figure 1** shows the flowchart of the present study. Importantly, we registered our review on Prospero (ID: CRD42024558280).

### Eligibility criteria

Given the novelty of this topic in the psychological scientific literature and its analysis across other areas (e.g., economy, law), we used two broad inclusion criteria: i) papers concerning problematic/excessive trading, regardless of the approach used to analyze the construct; ii) the construct has been analyzed theoretically (e.g., review papers, papers suggesting a theoretical model). Therefore, we also have a few broad exclusion criteria: i) papers about financial trading generally, even if analyzed from a psychological perspective; ii) empirical papers (unless the abstract reports the article also includes a theoretical section); iii) articles published in no-scientific journals.

#### Types of participants, outcome measures, comparison types, and study types

As this review deals with theoretical papers, we did not have restrictions concerning participants (e.g., age, gender, or nationality) or specific criteria concerning outcomes’ measure and comparison type. Finally, looking for theoretical papers, we included articles with no limitations concerning the authors’ language, country, or scientific area.

#### Search strategy

We searched for papers in six scientific databases: MEDLINE, PsycARTICLES, PsycINFO, PubMed, Scopus, and Web of Science (all years until April 22, 2024). More specifically, we used the search filters reported in Appendix A (including the filter adapted to Pubmed), without using field codes, and hence looking for the terms across the paper (not only in the title and/or abstract).

#### Study selection

Using the databases and search filter mentioned above, an author systematically searched the literature, identifying 4524 records, then evaluated for eligibility. As a first step, duplicates were removed (n = 1417), resulting in 3107 records being screened based on their titles and abstracts. Then, the author who took care of the search of the literature identified 20 theoretical papers to be further evaluated by a deep examination of their full text. Next, a second author analyzed a random set of 1200 excluded records, resulting in a 1% conflict between the two authors, which has been resolved – after a joint discussion of the research team – by including an additional 12 papers to the ones to be analyzed in full-text, agreeing to adopt a soften strategy about their inclusion/exclusion. Finally, the full-text screening resulted in 23 papers meeting the inclusion criteria. **Figure 1** shows the study selection process.

#### Process of data extraction and coding

We created a protocol to extract and encode the data from the full-text of the 23 papers that met the eligibility criteria. This protocol foresees the following variables: (1) authors and year of the publication; 2) reference to problematic trading as a phenomenon in itself – yes/no coding; 3) reference to specific features of trading (if problematic trading is described as a phenomenon in itself) – yes/no coding; 4) area of study of the authors (e.g., psychology, economy); 5) individual- or group-level analysis of problematic trading – individual and/or group coding; 6) criterion used to conceptualize trading as pathological (e.g., gambling criteria); 7) explicative variables suggested for problematic trading (e.g., overconfidence, personality traits).

## Results

The current systematic review is based on a total of 23 papers (reported in **Tables 1**, **2**) concerning problematic trading, equally distributed between the ones of psychological (12 papers) and other (11 papers) scholarly backgrounds. The studies analyzed covered the years between 1995 and 2024. The oldest papers are from scholars in the law/economy areas. The psychological literature on problematic trading began only a decade ago, namely in 2014, and almost all scholars adopted the behavioral addiction framework, usually assimilating problematic trading to gambling. In the no-psychology area, only the papers by Tierney (10) and Weidner (11) linked problematic trading to gambling, while the others did not provide a specific operationalization of the construct. The results are thoroughly presented in the following paragraphs, distinguishing between the studies of the no-psychological area (**Table 1**), where problematic trading first arose, and the psychological area (**Table 2** **).**

**Table 1:** Papers concerning problematic trading in no-psychology/health areas.

Authors/year	Country	Problematic Trading described as a phenomenon in itself	Reference to specific elements of trading	Area of study	Individual or group-level analysis	Criteria used to conceptualize trading as problematic	Explicative variables suggested
Bhattacharyya and Nanda (25)	USA	NO	NO	Economy	Individual	Impaired long-term performance and preference for short-term performance	Portfolio pumping
Corzo et al. (26)	Spain	NO	NO	Economy	Individual	Increase in trade volume	Overconfidence
De Bondt et al. (27)	Spain and USA	NO	NO	Economy	Individual	Not Available	Overconfidence; Inclination to choose among those issues that have already caught attention
Inghelbrecht and Tedde (28)	Belgium	NO	NO	Economy	Individual	Trading characterized by higher frequency and with higher quantity of money, and by a lower performance	Overconfidence (in the specific form of overestimation)
Kent and Hishleifer (29)	USA	NO	NO	Economy	Individual	Not Available	Overconfidence
Klhön (30)	Germany	NO	NO	Law/Economy	Individual/ Group	Trading too often after periods of high market returns, taking excessive risks, not learning or learning too little, failing to exit the market at the right time	Overconfidence; The constitution of financial markets influences investors’ overconfidence; Male investors trade more aggressively
Klontz et al. (31)	USA	NO	NO	Economy	Individual	Gambling criteria	Not Available
Mahoney (32)	USA	NO	NO	Law	Individual/ Group	Not Available	Irrationality; Incentive structures facing investors and financial intermediaries
Stout (33)	USA	NO	NO	Law/ Economy	Group	Not Available	Not due to irrationality: the investor trade because he/she believes that others in the market may be irrational
Tierney (10)	USA	NO	NO	Law	Individual	It might be a (“rational”) substitute for gambling	It might also be due to irrational belief; Gamified investing; Brokers with control over customer account might have incentives to increase their compensation (and trade excessively)
Weidner (11)	Germany	NO	NO	Sociology	Group	Not Available	The financial industry has addictive properties like the gambling industry, attracting risk-seeking individuals

**Table 2:** Papers concerning problematic trading in the psychological/health area.

Authors/year	Country	Problematic Trading described as a phenomenon in itself	Reference to specific elements of trading	Area of study	Individual or group-level analysis	Criteria used to conceptualize trading as problematic	Explicative variables suggested
Andrade and Newall (34)	UK	NO	NO	Psychology	Individual	Behavioral patterns previously observed in gamblers: Engaging in frequent, short-term trading and continuing despite experiencing losses; Chasing losses	Associated risks previously observed in gamblers: novelty seeking and low cooperativeness, sensation seeking, impulsiveness, market manipulation and scams
Arthur et al. (8)	Australia and Canada	YES	YES	Psychology	Individual	Trading characterized by addiction features (on its own – but they are not presented), but also as overlapped with gambling	Cognitive, motivational, and personality attributes similar to gamblers. *Cognitive features*: being overconfident in own investment skills, confirmation bias, illusion of knowledge and control over stock purchase outcome, preference for lottery-type stocks, high loss aversion. *Motivational feature:* leisure and “gambling” motives. *Personality features*: sensation seeking, risk-taking behavior
Delfabbro et al. (1)	Australia and Canada	YES	YES	Psychology	Individual	Behavioral addiction framework, plus reference to over-spending and compulsive checking	Over-estimations of the role of knowledge or skill, social learning and reinforcement, preoccupation/salience, fear of missing out (FOMO), anticipated regret
Grežo (35)	Slovakia	NO	NO	Psychology	Individual	Not Available	Overconfidence, with a distinction between indirect measures and three direct measures of overconfidence: overplacement, overestimation, and overprecision
Guglielmo et al. (36)	Italy	YES	YES	Psychology	Individual	Behavioral addiction framework, with particular reference to progressive loss of control over trading, tolerance and withdrawal symptoms. It is described as a maladaptive (persistent or recurrent) behavior related to trading that disrupts the family, personal and/or professional activities	Not Available
Håkansson et al. (37)	Sweden and Spain	NO	NO	Medicine/ Health Sciences	Individual	Gambling disorder criteria	Covid-19 as a factor that might increases trading frequency
Johnson et al. (38)	Australia	NO	NO	Psychology	Individual	Structure similarity with problematic gambling (i.e., excessive trading, chasing losses, borrowing money)	Demographic (young males) and personality (novelty seeking, low cooperativeness, motor impulsivity, FOMO) characteristics similar to share-traders and problem gamblers
Newall and Weiss-Cohen (9)	UK	NO	NO	Psychology	Individual	Gambling behavioral features: spending more time engaging in the activity and thinking about the activity more than wanted	Gambling psychological features: overconfidence, impulsiveness, sensation seeking
Roza et al. (39)	Brazil	NO	NO	Psychiatry	Individual	Gambling criteria	Online gambling risk factors: illusion of control, social learning and reinforcement, FOMO, chasing one’s losses
Sonkurt (6)	Turkey	NO	NO	Psychology	Individual	Gambling criteria	Not Available
Vismara et al. (40)	Italy and UK	YES	YES	Psychology/Medical Sciences	Individual	Urge-driven, compulsive checking and investing, and expansion of time dedicated to this activity, impairing all the domains of individual life. Presented as a digital type of Obsessive-Compulsive related disorder, but then also as overlapping with gambling features and highlighting its addiction features	Not Available
Wang (41)	China	NO	NO	Psychology	Individual	Income that is hardly enough to compensate for transaction cost	Blindy belief (i.e., overconfidence)

### The non-psychological literature concerning problematic trading

We included 11 papers in our review from the non-psychological area (see **Table 1**), which cover the years between 1995 and 2024. Most of these papers (seven) are from US scholars – with one involving both US and Spanish scholars – one from Spain, one from Belgium, and two from Germany. While seven research groups analyzed problematic trading from an individual perspective, two papers adopted a group-level analysis, and two offered a mixed perspective. In line with the topic of our review, which concerns a (problematic) financial behavior, the area of study – except for the sociologist Weidner (11) – is economy and law.

Remarkably, none of these studies conceptualized problematic trading as a phenomenon in itself by analyzing the specific elements of trading as a problematic/excessive behavior. In line with this, only three papers analyzed this problematic behavior through the lens of gambling (10, 11, 31), hence using gambling criteria to define trading as pathological. Klontz et al. (31) suggested ten gambling-based questions to be addressed to online traders, specifying that the person was moving from investing to gambling in case of positive answers. Similarly, Tierney (10) suggested that trading might be a rational substitute for gambling. Finally, Weidner (11) did not provide a specific criterion to define trading as problematic; however, he argued that some traders (both private and institutional) use the financial market to gamble.

Enlarging the results about the criteria used to define trading as problematic, these are not available for five (including 11) papers; two papers – previously described – refer to gambling (10, 31), while the remaining four used behavioral-based indicators. Bhattacharyya and Nanda (25) pointed out impaired long-term performance and preference for short-term performance. Corzo et al. (26) reported instead an increase in trade volume. Khlön (30) listed trading too often after periods of high market returns, taking excessive risks, not learning or learning too little, and failing to exit the market at the right time. Finally, Inghelbrecht and Tedde (28) referred to a trading behavior characterized by higher frequency and higher quantity of money, which is associated with lower performance. Their theoretical paper also includes research, and they found that their hypothesized lower performance was not present in their sample.

The no-psychological literature, however, gave rise to the field of study concerning the analysis of excessive/problematic trading as an irrational behavior, with most papers focusing on the role of overconfidence. Mahoney (32) noted two theoretical explanations for excessive trading in the literature. The noise trade model – which starts from excessive volatility – suggests that a subset of irrational traders confuse noise with information in the market. The heterogeneous expectation model by Stout (42) – which starts instead from excessive trading – posits that all investors are rational: all act based on the best information available (even if there might be a mistaken trader). Mahoney (32) suggests that even Stout (42) model should account for irrationality. He proposes that irrational trading exists and that excessive trading might result from the incentive structures facing investors and financial intermediaries (i.e., excessive trading could result from a conflict of interest between investors and intermediates whose compensation is based on transaction volume). Similarly, Tierney (10) also suggested that brokers with control over customer accounts might have incentives to increase their compensation (and trade excessively). However, Stout (33) reaffirmed that excessive trading is not due to irrationality. Possibly, the investor trades because he/she believes *others* in the market may be irrational.

Overconfidence is indicated as an explication variable of problematic trading by five studies (26, 28–30, 43), with Inghelbrecht and Tedde (28) particularly referring to overestimation, while De Bondt et al. (43) also reported the inclination to choose among those issues that have already caught attention. Additionally, Tierney (10), even if not explicitly referring to overconfidence, stated that (over)trading might be a “rational” substitute for gambling, but that for some people, it might be unintentional and due to irrational belief.

A few scholars have also addressed the role of financial markets structures in influencing problematic trading. Tierney (10) highlighted that gamified investing might incentivize excessive trading. Similarly, Weidner (11) pointed out that the financial industry has addictive properties like the gambling industry, attracting risk-seeking individuals. More generally, Klhön (30) suggested that the constitution of financial markets influences investors’ overconfidence, which he, along with other scholars, suggested contributes to excessive trading.

Finally, Klhön (30) pointed out that male investors trade more aggressively, while Bhattacharyya and Nanda (25) used portfolio pumping (that they defined as trading in the direction of the existing holdings of the risky asset to bolster the short-run measured value of the fund) as explication variable. For Klontz et al. (31), no explication variable arose from the reading of the paper.

### The psychological literature concerning problematic trading

The 12 papers included in this review from the psychological area (see **Table 2**) cover the years between 2014 and 2023. They were conducted in Western and Eastern countries: the UK, Australia, Canada, Italy, Slovakia, Sweden, Spain, Brazil, Turkey, and China. All of them adopted an individual-level study of problematic trading.

Interestingly, excluding the papers by Wang (41) and Grežo (35) – who did not describe problematic trading as a distinct phenomenon in itself nor associated it with gambling disorder – the other ten papers consistently adopted the behavioral addiction framework, usually referencing gambling disorder. Concerning Wang (41) and Grežo (35), in line with the lack of definition of problematic trading as a distinct diagnosis, there is not a transparent reference to the criteria used to define it as pathological (35), or there is just a reference to having an income that is hardly enough to compensate for transaction cost (41).

#### Problematic trading as a form of gambling

Among the ten papers that utilized the behavioral addiction framework, six did not present problematic trading as a separate entity. Instead, they suggested that it could be integrated into the concept of problematic gambling.

Three papers referred to gambling criteria to define trading as problematic (6, 37, 39). Håkansson et al. (37) advised including trading among the aspects to be analyzed with gambling tools. Sonkurt (6), in his case report of a cryptocurrency trader, used the DSM-5 (17) gambling criteria to evaluate the patient. Finally, Roza et al. (39) suggested evaluating patients with gambling-like behavior in trading by adapting the gambling criteria of the last edition of the Diagnostic and Statistical Manual of Mental Disorders (DSM-5 TR; 44). It is important to note that Roza et al. (39) wrote that there is not enough evidence currently supporting a distinctive diagnosis of problematic trading. Therefore, they considered that, in the future, there might be scientific evidence supporting the recognition of problematic trading as a disorder on its own.

The other three studies did not explicitly refer to gambling criteria to define trading as problematic but referred to behavioral features previously observed in gamblers. Andrade and Newall (34) cited engaging in frequent, short-term trading and continuing despite experiencing losses and the tendency to chase losses. Newall and Weiss-Cohen (9) similarly highlighted in problematic traders signs of behavioral dependency like gambling: spending more time engaging in the activity and thinking about the activity more than wanted. However, they also referenced previous studies that created scales specifically addressing problematic trading by resembling gambling instruments (45, 46). Finally, Johnson et al. (38), while suggesting cryptocurrency trading as a form of gambling, pointed out that the investment characteristics of cryptocurrency holders (i.e., excessive trading, chasing losses, borrowing money) suggest a structure similarity with problematic gambling.

#### Problematic trading as a disorder in its own right

Four research teams suggested conceptualizing problematic trading as a distinct diagnosis, hence analyzing the specific elements of trading (instead of merely proposing an overlap with gambling). More specifically, Arthur et al. (8) pointed out that besides considering problematic trading as a potential contributor to gambling, it is essential to also analyze it as a distinct phenomenon (and, more specifically, as a new behavioral addiction), especially concerning speculation. Roza et al. (39) previously suggested that financial investment and gambling are different activities that show considerable overlap, especially for financial speculation (e.g., day trading and cryptocurrency investing), as it is characterized by higher risk and shorter-term investments. Arthur et al. (8), instead, suggested that it is just speculation to be considered as a phenomenon on its own when compared to gambling as they posited that speculation is conceptually intermediate between investing and gambling (with whom hence share some features), whose specific qualities are related to time frame, expected returns, asset purchase, and economic utility. More specifically, speculation: i) has a variable time-frame (which is usually short for gambling and long for investment); ii) its expected returns are mixed and highly variable (while for gambling is usually negative and with low variability and for investment is usually positive and somewhat variable); iii) sometimes there is an asset purchase (which is absent in gambling and present in investment); iv) it is characterized by a mixed economic utility (while the economic utility is low for gambling and high for investing). The activities and instruments used in speculation are pretty distinct from gambling but less distinctive from investment. Moreover, the level of risk of speculation is high both in speculation and gambling (it is low for investment), while the role of chance is high across the three activities. Stakes are present in both speculation and gambling (not in investment). Finally, in gambling, there is a definitive event and outcome; in speculation, it is usually present (in investment, it is usually not present). However, Arthur et al. (8) did not present this last variable as a feature of speculation. In sum, Arthur et al. (8) conceptualized trading (in the specific form of speculation) as problematic by pointing out the need to recognize it as a contributor to problematic gambling (hence, to be assessed when evaluating gambling behaviors) but also as a new behavioral addiction in its own right (but they did not provide specific elements to definite it).

Similarly to Arthur et al. (2016), Delfabbro et al. (27) assimilated problematic trading – in the form of cryptocurrency trading – to online gambling and excessive social media use as sharing elements of risk involved in these two excessive behaviors. However, they also recognized its specific features: its 24-hour availability and long-form nature, the extreme volatility of outcomes, and the strong influence of sentiment and social influence. Therefore, regarding the criteria used to conceptualize trading as problematic, they adopted the behavioral addiction framework, with a specific reference to overspending and compulsive checking.

Guglielmo et al. (36) specifically stated to consider pathological trading, or “trading addiction,” as an (overlooked) form of behavioral addiction, pointing out the scant literature investigating addictive-like behavior among investors and criticizing the use of pathological gambling criteria or unspecified addiction criteria. They defined trading addiction as a persistent or recurrent maladaptive trading behavior that disrupts family, personal, and/or professional activities with associated progressive loss of control over trading, tolerance, and withdrawal symptoms (similar to substance use disorders). Moreover, they suggested (addiction-based) DSM-like pathological criteria specifically designed for trading, also considering that trading addiction might occur both in professional and non-professional traders. So, they proposed two criteria. The first criterion (A) posits persistent and recurrent problematic trading behavior (associated with impairment or distress) as indicated by at least five (up to 13) symptoms in the last 12 months. The list of symptoms includes, for example, tolerance, being restless or irritable when trying to cut down or stop trading, often trading when feeling distressed, and chasing losses. For some symptoms, Guglielmo et al. (36) pinpoint that the behavior should occur outside working hours when evaluating a professional trader. The second criterion (B) specifies that other psychological diagnoses should not better explain trading behavior. Finally, they also stressed that in their view, pathological trading reflects Goodman (47) concept of addiction, including both dependence and compulsion.

Finally, more recently, Vismara et al. (40) listed “compulsive online trading” as a type of digital form of Obsessive-Compulsive Related Disorders (OCRDs) while concurrently highlighting its overlap with gambling disorder and encouraging further studies on the topic to understand if it might be defined as an autonomous disorder, also considering its differences with gambling: stock exchange operations lie on individual knowledge and skills, and a longer-term perspective usually characterizes investment. Moreover, about the criteria used to define trading as problematic, they wrote that (online) trading is characterized by urge-driven, compulsive checking and investing in stock exchange transactions, with the consequent expansion of time dedicated to trading and consequent impairing of different functional areas. It is interesting to note that, even if Vismara et al. (40) included problematic trading among ORCDs, besides highlighting its overlap with gambling (i.e., a behavioral addiction), they also referenced the neuroimaging study by Knutson and Bossaerts (48) that supported the conceptualization of compulsive online trading as principally an addiction disorder, with the associated compulsive component as a second (additional) component.

#### Explicative variables of problematic trading

The psychological literature concerning problematic trading – regardless of its conceptualization as a disorder in its own right or as a form of gambling – highlighted some variables that might explain excessive/problematic trading. Among the 12 psychological papers included in our review, only three of them did not analyze these variables (6, 36, 40), while the other papers mainly focused on overconfidence (already studied in the non-psychological literature) and other risk factors previously observed in gamblers.

Cognitive risk factors similar to the ones previously observed in gamblers include being overconfident in own investment skills (8), having a confirmation bias – or only attending to information that confirms the person’s opinion (8), having an illusion of knowledge and control (8, 27, 39), preference for lottery-type stocks – that is, low-price stock with a slight chance of highly increasing their value (8), and high loss aversion (8), which might be conceptually linked to chasing ones’ losses, suggested by Roza et al. (39) as a risk factor. Moreover, Delfabbro et al. (27) considered preoccupation/salience, corresponding to continuously thinking about the activity. There are other scholars citing overconfidence (generally) as a risk factor for problematic trading (9, 41). Moreover, Grežo (35) provided a valuable contribution concerning this variable by distinguishing in his meta-analysis between indirect measures and three direct measures of overconfidence: overplacement (of the person’s abilities or performance relative to others), overestimation (of the person’s knowledge, abilities or performance compared to the actual performance), and overprecision (of the person’s ability to make accurate probability judgment). Regarding indirect measures, which showed a higher impact on financial decision-making, Grežo (35) underlined that some studies did not measure overconfidence but used excessive trading as a proxy. Concerning the three direct measures of overconfidence, overplacement showed a high effect, while overestimation only had a trivial effect, and overprecision was not statistically significant.

Regarding personality features, studies highlighted risk-taking behavior (8), impulsiveness (9, 34, 38), sensation seeking (8, 9, 34), novelty seeking and low cooperativeness (34, 38), and Fear of Missing Out (FOMO) – which, in the context of trading, consists in regretting not having made an investment or having sold in a specific period (27, 38, 39). Regarding FOMO, however, it should be noted that the study (49) included in Johnson et al (38) review showed that it is associated with an increased likelihood of cryptocurrency trading activity. However, it was not a predictor of problematic cryptocurrency trading.

Concerning motivational features, Arthur et al. (8) listed leisure (i.e., trading for fun and excitement) and “gambling” motives (i.e., aspiration of high payoffs and sensation seeking as the drivers). In this area, it is possible also to include anticipated regret (27), which cognitive psychology has recognized as a factor influencing many decisions (50, 51).

As additional (less analyzed) risk factors, Delfabbro et al. (27) and Roza et al. (39) highlighted the role of social learning and reinforcement, while Johnson et al. (38) suggested that young males are more at risk for problematic trading. Also, Andrade and Newall (34) pointed out market manipulation and scams as risk factors previously observed in gambling, while Håkansson et al. (37) suggested that COVID-19 (and similar crises) might increase trading frequency.

## Discussions

In the last decade, the psychological literature addressed problematic/excessive trading as a possible new clinical disorder deserving attention, usually adopting the behavioral addiction framework, with specific reference to Gambling Disorder. However, until now, no systematic review has been performed to organize the theoretical papers published so far across different areas of study to detect if there is a trading-specific related theorization and operationalization of the construct of problematic trading, which is a critical step for the analysis of new potential clinical disorders to avoid using aprioristic and confirmatory approach (16, 20).

Therefore, we performed a comprehensive search across six scientific databases (MEDLINE, PsycARTICLES, PsycINFO, PubMed, Scopus, and Web of Science), which led to the inclusion of 23 papers in our systematic review: 11 from the non-psychological literature and 12 from the psychological area. Interestingly, it arose that while the interest in problematic trading is recent in the psychological literature, it has received attention since the ‘90s in the economy/law area (32, 33). However, none of the 11 non-psychological papers conceptualized problematic trading as a phenomenon on its own. In line with this – in contrast with the behavioral addiction framework usually used in the psychological field – only three papers looked at the phenomenon assimilating it to gambling (10, 11, 31). Moreover, except for Klontz et al. (31) and Tierney (10), who used gambling criteria to define trading as problematic, the other papers did not provide criteria or used only behavioral-based indicators, like impaired long-term performance and preference for short-term performance or an increase in trade volume. Though, even if there is no evidence for the presence of a clear definition and theorization of problematic trading, the value of the non-psychological literature should be recognized, given that it gave rise to the analysis of excessive trading as an irrational behavior (32), where overconfidence plays a significant role (26, 28–30, 43).

Looking at the psychological literature – except for Wang (41) and Grežo (35) – the other ten papers consistently used the behavioral addiction context, usually assimilating problematic trading to problematic gambling. However, six papers did not describe problematic trading as a separate diagnosis from gambling. Three of them explicitly used gambling criteria (6, 37, 39), while other three studies used behavioral features previously observed in gamblers, such as engaging in frequent, short-term trading, the tendency to chase losses, and thinking about the activity more than wanted (9, 34, 38). However, four papers conceptualized instead problematic trading as a distinct diagnosis, considering the specific elements of financial trading. Arthur et al. (8) underlined that it is speculation to be a behavioral addiction on its own, as it is conceptually intermediate between investing and gambling. Delfabbro et al. (27), focusing on cryptocurrency trading, defined it as similar to (online) gambling and excessive social media use but also as having specific features. Vismara et al. (40), even if they included problematic trading among ORCDs, highlighted its overlap with gambling and suggested that previous research showed that it is principally an addiction disorder, with the associated compulsive component as a second (additional) component. Finally, Guglielmo et al. (36) introduced “trading addiction” as an (overlooked) form of behavioral addiction, and they suggested DSM-like addiction-based criteria specifically designed for trading, also specifying that trading addiction might occur both in professional and non-professional traders.

In sum, the psychological literature seems to begin supporting the conceptualization of problematic trading as a disorder on its own, usually assimilated with gambling – even when recognizing its specific features – also considering the explicative variables analyzed for this problematic behavior, that usually refer to gambling risk factors, such as personality traits and cognitive bias already demonstrated in gambling. Thus, we conclude that there is no evidence for a definition of problematic trading that considers its specific pinpoints, as it has been usually adopted aprioristically the gambling framework.

In line with the advice from Billieux et al. (16) and Kardefelt-Winther (20), future studies should analyze problematic trading as a phenomenon on its own, avoiding the aprioristic use of the gambling framework, to unveil the real nature and features of this new potential clinical disorder. Later, it will be possible to introduce its definition and operationalization and a proper measurement instrument. In this vein, it would be helpful to perform qualitative studies that allow an in-depth knowledge of the phenomenon (besides quantitative studies). Dixon et al. (52) performed a qualitative analysis on 13 stock traders divided into two focus groups (i.e., regular or occasional traders). They focused on the manifestations and consequences of excessive trading and its relationship with gambling disorder. So, it would be critical to expand this line of research further by investigating the phenomenology of problematic trading, possibly avoiding reference to gambling and avoiding a confirmatory approach also in qualitative studies.

## Conclusions

We should note some limitations of the present systematic review. First, even if we conducted it rigorously, the (low) percentage of disagreement that arose between the two authors during the check of a random sub-sample of the excluded records in the title/abstract screening phase suggests that some papers – potentially inherent to the review – could have been excluded. We speculate that this might apply mainly to the non-psychological literature since (as reported before) it generally lacks a specific definition and/or reference to problematic/excessive trading, increasing the likelihood of being excluded based on the reading of their abstract. Also, even if we used databases including different scholarly areas, we did not use financial-specific databases, relying mainly on general databases (i.e., Scopus and Web of Sciences) or psychology/health databases (i.e., MEDLINE, PsycARTICLES, PsycINFO, and PubMed).

Despite these limitations, we highlight that our search has been conducted on all the fields (not only on title and abstract), hence enlarging the possibility of gathering papers that do not explicitly refer to problematic trading. Also, this is the first systematic review that does not focus on the links between gambling and trading but tries to systematize the theoretical views concerning problematic trading, also outside the psychological area. Through this effort, we underlined that the literature – despite a few scholars proposing to conceptualize it as a behavioral addition on its own – currently lacks (both in the psychological and non-psychological area) a definition and operationalization of problematic trading that is based on a comprehensive analysis of its specific features. Thus, we encourage performing qualitative studies to gain a deep insight into problematic trading features, which can then be used to suggest a definition of this new potential clinical disorder without using a gambling-based aprioristically perspective.
